# Survey of internal parasites in captive ungulates from Lahore parks, Pakistan

**DOI:** 10.2478/helm-2025-0016

**Published:** 2025-09-30

**Authors:** S. Irum, M. Faiz, K. Aftab, K. Aftab, Mehjabeen, H. Zulqarnain, S. Gondal

**Affiliations:** Department of Zoology, University of Gujrat, Gujrat 50700, Pakistan

**Keywords:** parasites, GIT infection, captive wildlife ruminants, zoological parks

## Abstract

Wild animals are mostly kept for recreational purposes in zoos and parks. Health-related issues faced by these enclosure animals. The GIT (gastrointestinal tract) of an animal is affected by parasitic infections. The current study was conducted to fi nd out the prevalence and diversity of GI parasites of wild animals enclosed in the different parks and zoos of Lahore. Fresh faecal samples (n=110) of captive ungulate species were collected from Lahore Zoo (n=55), Jallo Park (n=35) and the woodland wildlife park (n=20). Samples obtained from captive ungulates include 23 from black buck, 24 from moufl on sheep, 30 from hog deer, 13 from nilgai and 20 from urial. Methods such as the fl otation concentration technique, the sedimentation technique and the faecal egg count per gram were used to analyze the diversity of endoparasites in faecal samples. Parasitological analyses detect 10 parasites in the faecal sample. Kruskal-Wallis Post Hoc statistical test was used to compare the prevalence of the specifi ed parasitic species among different captive ungulates. The results show that individuals of lower age are more susceptible to infections. The overall diversity of GI parasitic infections in captive ungulates was 77.25 %. Survivability of captive ungulates could be affected by the prevalence of parasitic species. These fi ndings can be used to develop effective health protocols for captive ungulates, thereby reducing the severe consequences of GI parasitic infections in captive wild animals.

## Introduction

Surveying and diagnosing internal parasites of captive ruminants is critical for maintaining overall animal health and ensuring the species’ productivity within various captive management strategies. Faecal parasite identifi cation also gives economic benefi ts (Pauling *et al*., 2016).

Pakistan is rich in biodiversity, notably in the arid and semi-arid regions, which account for over 80 % of the total geographical area. A variety of animal and plant species are vulnerable and/or endangered, primarily due to habitat loss and parasitism (Baig & Al-Subaiee, 2009). The protection of endangered species is a serious and emerging issue in Pakistan. Also, agencies like Pakistan Wildlife Foundation, WSPA Pakistan, WWF Pakistan, Wildlife sanctuaries, zoological gardens and wild parks have been established to protect threatened species. Moreover, wildlife departments at the provisional level, e.g., the Punjab wildlife department, have been established to protect endangered and threatened species (Shah *et al*., 2024). The current study examined two vulnerable, two least count and one endangered species of Lahore-Punjab captive areas: Lahore Zoo, Lahore Safari Park and Jallo Wildlife Park. The five species appear on the Red List of endangered, threatened and vulnerable species of the International Union for Conservation of Nature (IUCN). According to the list, *Antilope cervicapra* (black buck) is categorized as least count (LC), *Axis porcinus* (hog deer) categorized as endangered (EN), *Ovis gmelini* (mouflon sheep) is categorized as vulnerable(VU), *Boselaphus tragocamelus* (nilgai) as least count (LC), and *Ovis vignei* (urial) categorized as vulnerable (VU). Wildlife strategies use four main approaches to address underlying social, personal, and environmental factors that limit people’s capacities, resources, and abilities to attain and maintain health improvements (Stephen, 2022). These four strategies include developing health and management policies that safeguard the determinants of wildlife health, emphasizing the creation of healthy environments that facilitate long-term access to the determinants of health, enhancing teamwork and shifting the focus of wildlife health from disease management alone to a continuum of care. Reducing vulnerability, minimizing chronic harms, fostering recovery from actualized harms, and preserving health capacity are all part of this continuum of care (Stephen, 2022). Parks and reserves have been established to protect endangered animals across the country. However, animals present in semi-captivity require control of health management, especially with regard to viral and parasitic disorders (Aissa *et al*., 2021).

Antelopes are grazing ruminants, so they easily become susceptible to internal parasite illnesses such as gastrointestinal and blood parasites. Parasites and hosts coexist in nature in a delicate equilibrium. Intense parasitism can significantly alter wildlife host populations, including reproduction, survival, growth, and behaviour (Aissa *et al*., 2021).

The study by Rana *et al*. (2015) indicated that *Stronglyoides* (82.55 %), *Trichostrogylus* (81.39 %), and *Trichuris* (74.41 %) have shown maximum prevalence, followed by *Haemonchus* (32.55 %), *Paramphistomum* (31.39 %), and *Moniezia* (6.98 %), respectively, in hog deer. (Ahmed, 2020) study indicated on urial internal parasites prevalence that is *Moniezia* (9.6 – 4 %), *Trichuris*. (41.5 %) and *Strongyloides* (7.58 %). Prevalence of *Moniezia, Trichuris and Strongyloides* in mouflon was 6.5 %,0.9 % and 43 % (Kapnisis *et al*., 2022). In black buck prevalence of *Moniezia* (14 %) among cestode; *Strongyloides* (16 %) and *Trichuris* (6 %) among nematodes (Chaudhary & Maharjan, 2017). In nilgai prevalence of Strongyloides (3.92 %)(Shirbhate & Shirbhate, 2022).

Keeping in view the importance of antelopes for conservation, the present study was designed to check the prevalence of internal parasites and means comparison of internal parasites between different ungulates in individuals kept in zoological parks.

## Materials and Methods

### Sampling methodology

A cross-sectional study design was followed to conduct research. Quantitative research methods were used to identify the prevalence of GI parasites. One hundred ten fresh faecal samples were collected from 5 captive wildlife species by using the epitool. Fresh faecal samples were collected from 5 antelope species having no medical (illness) history. Faecal samples were collected in the early morning. In order to protect samples from contamination, sanitized polystyrene spatulas were used to collect samples from the ground. Samples were placed in a plastic container (having 8 % formalin). To ensure safety during transport, the plastic container was placed in a biohazard plastic bag. The open ends of the plastic biohazard bags were closed tightly and labelled with a marker according to the species of antelope. These samples were quickly brought to the University of Gujrat Parasitology Lab.

### Study area and animals

**Fig. 1.**
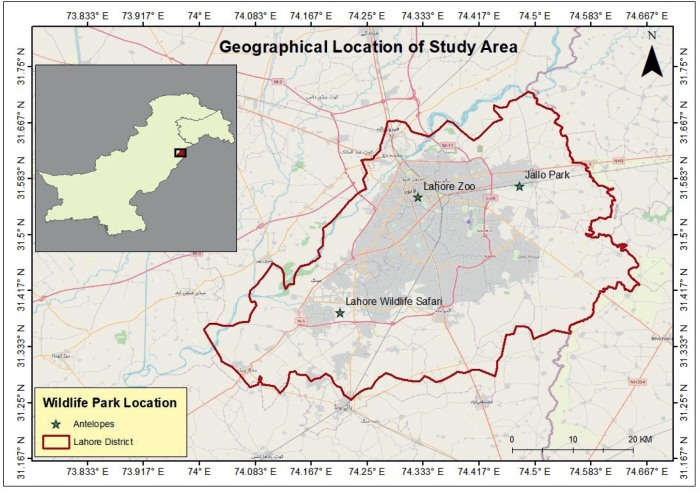
Geographical Location of Wildlife Park.

The research was conducted from April 2023 to November 2023 in three captive areas, namely Lahore Zoo, Lahore Safari Park, and Jallo Wildlife Park (Fig. 1). These areas were selected due to the availability of Hog deer (an antelope species) in Pakistan. Faecal samples of 110 antelopes, including both males and females, were randomly collected from these three captive areas to study GIT endoparasites.

Lahore is situated in the northeast part of the country, lies on the river Ravi and is about 24 kilometers (15 miles) from the Wagah Border. It also experiences four distinct seasons, including a lengthy, sweltering summer, a dry, chilly winter, a monsoon, and dust storms. Extreme weather occurs in May, June, and July when the temperature is between 40 and 48 °F (104 and 118°F). The beginning of the monsoon season is marked by Lahore’s record-breaking rainfall of 221 millimeters (8.7 in) on August 13, 2008 (Imran & Mehmood, 2020).

Fresh faecal samples (n=110) were collected from Lahore Zoo (n=55), Jallo Park (n=35), and the woodland wildlife park (n=20). Samples obtained from antelopes include black buck (n=20), mouflon sheep (35), hog deer (19), nilgai (23) and urial (13).

### Laboratory analyses

The faecal samples were analyzed through sedimentation techniques, flotation techniques and eggs per gram for identification of parasites. In floatation concentration techniques, faecal samples weighing a total of 1 gram were placed in a beaker along with 15 ml of flotation solution, which was thoroughly agitated. A 15ml tube was used to contain the solution after it had been filtered using a tea strainer. The test tube was gently covered with a cover slip and set in a test tube rack with a convex meniscus. Give the test tube 15 to 20 minutes to stand. Following that, a clean glass slide was used to mount the cover slip, and it was examined under a microscope (4X, 10X, and 40X) (Soares *et al*., 2024).

Sedimentation technique was performed by properly combining 3 grams of faeces with 40 – 50 ml of tap water in a beaker. A tea filter was then employed to strain the mixture. The solution was poured into a test tube for duration of five minutes. Delicately pour out the liquid that is above the sediment, and then mix the solution again by adding 5ml of water. The operation was repeated 3 to 5 times till the supernatant became clear. Next, a sediment sample was carefully deposited onto a clean glass slide. A cover slip was placed on a glass slide under a microscope (Rojas-Moncada *et al*., 2024).

Microslides generated from sedimentation and flotation samples were examined under a microscope. Quantitative McMaster Technique, a saturated sugar solution with a specific gravity of 1.2, was added to each sample of faeces, which weighed 2 grams, before it was filtered and put on a McMaster slide (Rojas-Moncada *et al*., 2024).

### Statistical analyses

The data acquired throughout the research trials will be statistically analyzed using SPSS version 25. This analysis will include Posthoc Kruskal-Wallis tests for repeated measurements (van Galen *et al*., 2023).

## Ethical Approval and/or Informed Consent

Formal permission was taken from the parks and zoos administration to collect samples from antelopes while ensuring their safety. Ethical approval was obtained from the Department of Zoology of the University of Gujrat. This research was approved by research ethics committee (REC) at Gujrat University. T

## Approved Experimental Protocol

Laboratory tests were performed to check the quantity of eggs present in faeces. Solusbys’ methodology is an approved experimental protocol to count faecal eggs per gram.

## Results

To see the prevalence of gastrointestinal parasites, 110 faecal samples were collected at Lahore parks and zoos. Out of 110 samples, parasitic prevalence was examined in 77.25 % of the samples. Hog deer represented the highest percentage at 28.2 %, mouflon sheep and black buck follow with 20 %, urial follow with 18.2 % and nilgai were the least frequent at 13.6 %. Prevalence of parasitic infection varies across different groups, as shown in Table. 3.

Data is not normally distributed. A Kruskal-Wallis test was performed to compare parasitic loads among various antelope species. P-values show that the trends of parasitic species vary among antelope species according to age, as shown in Table 1. To check which parasitic species prevalence varies significantly in antelopes species, a post hoc test was performed, as shown in Table 2. *Trypanosoma, Paramphistomum, Haemonchus*, and *Moniezia* p-values show that the diversity of these four parasites was similar across all antelope species (black buck, urial, hog deer, mouflon sheep, and nilgai). The p-values for *Theileria, Trypanosoma, Trichuris, Strongyloides*, and *Babesia* indicate statistically significant differences in prevalence among antelope species. In comparison to other species, Nilgai had a significantly lower level of *Theileria, Trypanosoma* and *Babesia. Trichuris* had a higher prevalence in hog deer. *Strongyloides* were observed at higher levels in nilgai and mouflon sheep. Parasitic species with high prevalence in the study were *Trypanosoma* parasites (38.18 %), *Moniezia* (27.27 %), *Paramphistomum* (24.55 %), *Trichuris* (22.73 %), *Haemonchus* (20.91 %), *Babesia, Moniezia, Strongyloides* (19.09 %), *Theileria* (13.64 %) and *Trypanosoma* (12.73 %) respectively. These results pointed to the differences in parasitic loads among the studied species and called for religious consideration of aspects such as habitat, diet, or immunity where such differences are being observed. Hence, defining these differences is important to come up with appropriate steps that need to be taken when it comes to parasite control, as well as for the management and conservation of some of the wildlife species.

**Table 1.** Kruskal-Wallis test to compare the prevalence of the specified parasitic infections among different antelopes species.

	T.	T.S.	B.	T.A.	M.A.	M.B.	T.G.	S.P.	P.C.	H.C.
**Chi-Square**	2.178	14.025	3.085	0.565	7.185	0.561	1.856	6.801	1.547	4.467
**Df**	2	2	2	2	2	2	2	2	2	2
**Asymp. Sig**.	0.337	0.001	0.214	0.754	0.028	0.756	0.395	0.033	0.461	0.107

* Dependent Variable: Antelopes Species

**Table 2.** Post-hoc test for comparison of parasitic burden among various antelopes species.

Dependent Variable	(I) species	(J) species	Mean Difference (I-J)	Std. Error	Sig.
***Trypanosoma***	black buck	urial	-0.177	0.151	0.768
		hog deer	-0.050	0.137	0.996
		mouflon sheep	-0.227	0.148	0.540
		nilgai	-0.127	0.164	0.937
	urial	black buck	0.177	0.151	0.768
		hog deer	0.127	0.140	0.894
		mouflon sheep	-0.050	0.151	0.997
		nilgai	0.050	0.167	0.998
	hog deer	black buck	0.050	0.137	0.996
		urial	-0.127	0.140	0.894
		mouflon sheep	-0.177	0.137	0.692
		nilgai	-0.077	0.154	0.987
	mouflon sheep	black buck	0.227	0.148	0.540
		urial	0.050	0.151	0.997
		hog deer	0.177	0.137	0.692
		nilgai	0.100	0.164	0.973
	nilgai	black buck	0.127	0.164	0.937
		urial	-0.050	0.167	0.998
		hog deer	0.077	0.154	0.987
		mouflon sheep	-0.100	0.164	0.973
***Theileria***	black buck	Urial	-0.014	0.103	10.000
		hog deer	0.072	0.093	0.937
		mouflon sheep	0.091	0.100	0.893
		nilgai	-0.264	0.111	0.131
	urial	black buck	0.014	0.103	10.000
		hog deer	0.085	0.095	0.897
		mouflon sheep	0.105	0.103	0.846
		nilgai	-0.250	0.113	0.186
	hog deer	black buck	-0.072	0.093	0.937
		urial	-0.085	0.095	0.897
		mouflon sheep	0.019	0.093	10.000
		nilgai	-0.335^*^	0.104	0.015
	mouflon sheep	black buck	-0.091	0.100	0.893
		urial	-0.105	0.103	0.846
		hog deer	-0.019	0.093	10.000
		nilgai	-0.355^*^	0.111	0.016
	nilgai	black buck	0.264	0.111	0.131
		urial	0.250	0.113	0.186
		hog deer	0.335^*^	0.104	0.015
		mouflon sheep	0.355^*^	0.111	0.016
***Babesia***	black buck	urial	0.227	0.118	0.310
		hog deer	0.098	0.106	0.887
		mouflon sheep	-0.045	0.115	0.995
		nilgai	-0.173	0.128	0.660
	urial	black buck	-0.227	0.118	0.310
		hog deer	-0.129	0.110	0.764
		mouflon sheep	-0.273	0.118	0.149
		nilgai	-0.400^*^	0.130	0.023
	hog deer	black buck	-0.098	0.106	0.887
		urial	0.129	0.110	0.764
		mouflon sheep	-0.144	0.106	0.661
		nilgai	-0.271	0.120	0.168
	mouflon sheep	black buck	0.045	0.115	0.995
		urial	0.273	0.118	0.149
		hog deer	0.144	0.106	0.661
		nilgai	-0.127	0.128	0.857
	nilgai	black buck	0.173	0.128	0.660
		urial	0.400^*^	0.130	0.023
		hog deer	0.271	0.120	0.168
		mouflon sheep	0.127	0.128	0.857
***Moniezia***	black buck	urial	-0.205	0.118	0.420
		hog deer	-0.277	0.107	0.078
		mouflon sheep	0.000	0.115	10.000
		nilgai	-0.221	0.128	0.422
	urial	black buck	0.205	0.118	0.420
		hog deer	-0.073	0.110	0.964
		mouflon sheep	0.205	0.118	0.420
		nilgai	-0.017	0.131	10.000
	hog deer	black buck	0.277	0.107	0.078
		urial	0.073	0.110	0.964
		mouflon sheep	0.277	0.107	0.078
		nilgai	0.056	0.120	0.990
	mouflon sheep	black buck	0.000	0.115	10.000
		urial	-0.205	0.118	0.420
		hog deer	-0.277	0.107	0.078
		nilgai	-0.221	0.128	0.422
	nilgai	black buck	0.221	0.128	0.422
		urial	0.017	0.131	10.000
		hog deer	-0.056	0.120	0.990
		mouflon sheep	0.221	0.128	0.422
***Strongyloides***	black buck	urial	0.045	0.113	0.994
		hog deer	-0.116	0.102	0.787
		mouflon sheep	-0.364^*^	0.110	0.011
		nilgai	-0.355^*^	0.123	0.037
	urial	black buck	-0.045	0.113	0.994
		hog deer	-0.161	0.105	0.541
		mouflon sheep	-0.409^*^	0.113	0.004
		nilgai	-0.400^*^	0.125	0.015
	hog deer	black buck	0.116	0.102	0.787
		urial	0.161	0.105	0.541
		mouflon sheep	-0.248	0.102	0.116
		nilgai	-0.239	0.115	0.239
	mouflon sheep	black buck	0.364^*^	0.110	0.011
		urial	0.409^*^	0.113	0.004
		hog deer	0.248	0.102	0.116
		nilgai	0.009	0.123	10.000
	nilgai	black buck	0.355^*^	0.123	0.037
		urial	0.400^*^	0.125	0.015
		hog deer	0.239	0.115	0.239
		mouflon sheep	-0.009	0.123	10.000
***Paramphistomum***	black buck	urial	0.082	0.133	0.972
		hog deer	-0.076	0.120	0.969
		mouflon sheep	-0.182	0.130	0.628
		nilgai	-0.152	0.144	0.830
	urial	black buck	-0.082	0.133	0.972
		hog deer	-0.158	0.123	0.703
		mouflon sheep	-0.264	0.133	0.281
		nilgai	-0.233	0.147	0.508
	hog deer	black buck	0.076	0.120	0.969
		urial	0.158	0.123	0.703
		mouflon sheep	-0.106	0.120	0.904
		nilgai	-0.075	0.135	0.981
	mouflon sheep	black buck	0.182	0.130	0.628
		urial	0.264	0.133	0.281
		hog deer	0.106	0.120	0.904
		nilgai	0.030	0.144	10.000
	nilgai	black buck	0.152	0.144	0.830
		urial	0.233	0.147	0.508
		hog deer	0.075	0.135	0.981
		mouflon sheep	-0.030	0.144	10.000
***Haemonchus***	black buck	urial	-0.009	0.125	10.000
		hog deer	-0.199	0.113	0.398
		mouflon sheep	-0.136	0.122	0.797
		nilgai	-0.242	0.136	0.386
	urial	black buck	0.009	0.125	10.000
		hog deer	-0.190	0.116	0.476
		mouflon sheep	-0.127	0.125	0.847
		nilgai	-0.233	0.138	0.446
	hog deer	black buck	0.199	0.113	0.398
		urial	0.190	0.116	0.476
		mouflon sheep	0.063	0.113	0.981
		nilgai	-0.043	0.127	0.997
	mouflon sheep	black buck	0.136	0.122	0.797
		urial	0.127	0.125	0.847
		hog deer	-0.063	0.113	0.981
		nilgai	-0.106	0.136	0.935
	nilgai	black buck	0.242	0.136	0.386
		urial	0.233	0.138	0.446
		hog deer	0.043	0.127	0.997
		mouflon sheep	0.106	0.136	0.935

**Table. 3:** Prevalence of internal parasites in captive ungulates.

Prevalence					
Parasites	Black buck	Urial	Hog deer	Mouflon Sheep	Nilgai
***Trypanosoma***	27	45	32	50	40
***Theileria***	13.63	15	6.45	4.54	40
***Babesia***	22.72	0	12.90	27.27	40
***Moniezia***	18.18	10	0	4.54	46.66
***Trichuris***	4.54	25	32.25	4.54	26.66
***Strongyloides***	9.09	35	45.16	27.27	6.66
***Paramphistomum***	9.09	10	58.06	9.09	6.66
***Haemonchus***	4.54	0	16.12	40.90	40
**Total**	108%	140%	201%	165%	244%

## Discussion

The present research aimed to elucidate the diversity of gastrointestinal parasites and estimate worm burden in captive ungulates, including Wild Hog Deer, Wild Urial, Wild Mouflon Sheep, Nilgai, and Black Buck. Microscopic examination shows that 77.25 % of samples had parasitic prevalence. Out of different ungulate families, the frequency distribution revealed that Hog deer constituted the largest proportion of the sampled population to the tune of 28 percent. These results aligned with research carried out by Kutz *et al*. (2001), with a high proportion of population surveys containing Hog deer.

The highest prevalence of internal parasites is in Nilgai, followed by Hog deer, Mouflon Sheep, Urial, and Black buck, respectively. This result aligns with a study conducted by Naz *et al*. (2021), which found that the overall highest prevalence was recorded in nilgai, followed by urial (*O. orientalis*) and blackbuck (*A. cervicapra*) (Naz *et al*., 2021). In hog deer, the prevalence of *Stronglyoides, Trichuris, Paramphistomum*, and *Moniezia* is 45 %, 32 %, 58 %, and 0 %, respectively. This result aligned with research carried out by Rana *et al*. (2015) that *Stronglyoides* (82.55 %), *Trichostrogylus* (81.39 %) and *Trichuris* (74.41 %) have shown maximum prevalence, followed by *Haemonchus* (32.55 %), *Paramphistomum* (31.39 %) and *Moniezia* (6.98 %), respectively, in hog deer. In urial prevalence of *Moniezia, Trichuris*, and *Strongyloides* are 10 %, 25 % and 35 %, respectively. This result is in accordance with research carried out by Ahmed (2020) on the prevalence of urial internal parasites, namely *Moniezia* (9.6 – 4 %), *Trichuris* (41.5 %), and *Strongyloides* (7.58 %). The prevalence of *Moniezia, Trichuris*, and *Strongyloides* is 4 %, 4 %, and 27 %, respectively, which aligns with research indicating that the prevalence of *Moniezia, Trichuris*, and *Strongyloides* in mouflon is 6.5 %, 0.9 %, and 43 % (Kapnisis *et al*., 2022). In black buck, the prevalence of *Moniezia, Strongyloides*, and *Trichuris* is 18 %, 9 %, and 4 %, respectively, which aligns with *Moniezia* (14 %) among cestodes; *Strongyloides* (16 %) and *Trichuris* (6 %) among nematodes (Chaudhary & Maharjan, 2017). In nilgai, the prevalence of *Strongyloides* is 6 % which aligns with the prevalence of *Strongyloides* (3.92 %) (Shirbhate & Shirbhate, 2022).

The difference in the rates of parasitic infestations was found to vary between the different families of the antelope. Hog deer had the highest infection indices for the majority of the parasites, especially *Trichuris globulosa* and *Moniezia benedeni*. This result concurs with the findings of von Dohlen *et al*. (2017), which report the highest infection rate in hog deer. Nilgai had moderate to high infection denseness in different parasites including *Theileria schizonts, Babesia*, and *Trypanosoma* amastigote conforming to the study of Ezenwa (2004) in ruminants.

The highest prevalence of parasitic infection caused by *Trypanosoma* in antelopes was observed, which is in agreement with a study carried out by the authors Kutz *et al*. (2001) with a high prevalence of *Trypanosoma* in the wild ruminant populations.

## Conclusions

The study reported that 77.25 % of the sample had parasitic prevalence in zoological parks across Lahore. It also identifies that antelopes appearing healthy had a high prevalence of GIT and were mostly effected by endoparasites. The highest prevalence of parasitic species caused by *Trypanosoma* parasites (38.18 %), *Moniezia benedeni* (27.27 %), *Paramphistomum cervi* (24.55 %), *Trichuris globulosa* (22.73 %), *Haemonchus contortus* (20.91 %), *Babesia, Moniezia expansa, Strongyloides papillosus* (19.09 %), *Theilria* schizonts (13.64 %) and *Trypanosoma* amastigote (12.73 %), respectively. The study also highlights that poor deworming protocols and management in zoological parks for wild ruminants. The high prevalence further shows that there is a need for continuous monitoring as well as vigorous control measures to minimize the impact of parasitic diseases on wildlife and the related conservation efforts. Moreover, it is necessity for administrations to improve the quality of food and hygiene conditions for captive ruminants. Future study should be conducted on types of environments which enhance immunity and suit the well-being of the antelope species.
